# 22533 Marshallese Mothers' and Maternal Health Care Providers' Perspectives of the Structural and Socio-Cultural Barriers to Prenatal Care: A Comparison Article

**DOI:** 10.1017/cts.2021.616

**Published:** 2021-03-31

**Authors:** Britni Ayers, Lauren Haggard-Duff, Pearl McElfish

**Affiliations:** University of Arkansas for Medical Sciences Northwest

## Abstract

ABSTRACT IMPACT: This study will be used to culturally tailor interventions to reduce maternal and infant health disparities in a Marshallese community. OBJECTIVES/GOALS: Inadequate prenatal care is associated with adverse birth outcomes including preterm births, low birth weight infants, and neonatal mortality. Marshallese Pacific Islanders are less likely to receive early and consistent prenatal care compared to other racial/ethnic groups and are thus at a higher risk for maternal and infant health disparities. METHODS/STUDY POPULATION: This article used a qualitative comparative analysis method to compare and contrast the perceived barriers to prenatal care for the prospective of Marshallese mothers and Maternal Health Care Providers (MHCPs). RESULTS/ANTICIPATED RESULTS: Marshallese mothers and MHCPs identified the same structural barriers to prenatal care: health insurance, transportation, and language. The socio-cultural barriers to prenatal care were depicted quite differently by Marshallese mothers verses MHCPs. DISCUSSION/SIGNIFICANCE OF FINDINGS: While the description of structural barriers were consistent among Marshallese mothers and MHCPs, the socio-cultural barriers and the value assigned to those barriers was quite different. Understanding the perspectives from both lenses is an important step towards addressing the barriers to prenatal care among Marshallese.

